# “I ask them what autism means for them”: a qualitative study of staff experiences of working with autistic women and birthing people in community perinatal mental health teams

**DOI:** 10.1186/s12888-025-07497-6

**Published:** 2025-10-27

**Authors:** V. Westgate, C. Thompson, D. Caramaschi, H. O’Mahen

**Affiliations:** 1 Department of Psychology, University of Exeter, Exeter, UK; 2 Population Health Sciences Institute, University of Newcastle, Newcastle upon Tyne, UK

**Keywords:** Mental health, Perinatal, Qualitative research, Mental health services, Autism

## Abstract

**Background:**

Autistic women and birthing people (AWBP) face an increased risk of perinatal mental health issues and may be referred to Community Perinatal Mental Health Teams (CPMHTs). However, staff members of CPMHTs often lack sufficient training and knowledge about autism, leading to barriers in providing appropriate care. To date, no studies have examined the experiences of staff supporting autistic women and birthing people within CPMHTs.

**Methods:**

We carried out individual qualitative interviews with 20 staff members from different professional backgrounds across four CPMHTs in England to understand their experiences working with AWBP and to identify how CPMHTs could better meet their needs. Thematic analysis was used to synthesise findings.

**Results:**

We identified four key themes: (1) recognizing the complexities of AWBP, (2) addressing the unique needs of AWBP (3) varying levels of knowledge and understanding across both the CPMHTs and individual clinicians, and (4) envisioning improvements for the future. Staff reported that working with AWBP was an unexpected aspect of their roles, further complicated by difficulties in accessing autism assessments for those who had not yet received a formal diagnosis. They highlighted both barriers to providing appropriate care and the adjustments they could make to improve their practice. Additionally, staff expressed a need for targeted training and suggested other areas for development, including appointing an autism champion, employing autistic peer support workers, and involving individuals with lived experience of autism and perinatal mental health challenges in co-producing service improvements.

**Conclusions:**

Working with AWBP is a current area of challenge for CPMHTs. Perinatal-focused autism training and expert supervision are important for staff to feel confident in making appropriate adaptions to care to provide the best possible support.

**Supplementary Information:**

The online version contains supplementary material available at 10.1186/s12888-025-07497-6.

## Background

Autistic women and birthing people (AWBP) are at an increased risk of experiencing perinatal mental health issues [1]. However, research has shown that clinicians in adult healthcare settings, including mental health services, often have limited training or knowledge about autism, despite providing care for autistic individuals [2, 3] and autistic individuals themselves face significant barriers to accessing mental healthcare [4, 5]. A number of recent systematic reviews, including our own work, draw attention to the unique challenges of being autistic during the perinatal period, for example: the sensory demands of pregnancy and having a new baby, accessing healthcare services which are designed for neurotypical people, executive functioning differences around managing an infant’s routine, and the impact on a parent’s mental health [6–13]. However, there is a gap in the literature regarding the experiences of professionals who support the mental health of AWBP during the perinatal period.

Autism is a neurodevelopmental condition characterised by challenges with social communication, social interaction and repetitive and restrictive behaviour, often alongside sensory processing differences [14]. Autistic people experience a high incidence of mental health problems. Martini et al.’s cohort study found that 64.7% of autistic women included in their analysis had at least one psychiatric diagnosis by age 25 [15]. This contributes to increased mortality as evidenced by research that found autistic women to be 13 times more likely than non-autistic women to die by suicide [16].

Autistic people face more healthcare inequalities due to challenges such as making appointments by telephone, communicating with clinicians due to the double empathy problem (which suggests that communication breaks down between autistic and non-autistic people due to mutual misunderstanding [17]), and struggles with the waiting room environment due to individual sensory needs [18]. In addition, lack of knowledge or training within healthcare providers can contribute to difficulties in understanding autistic people’s needs, onward referral, and appropriate treatment planning [4, 19]. For instance, accessing psychological therapy in particular can be obstructed by lack of therapist expertise or willingness to tailor approaches to support the needs of autistic people [20]. Clinicians also report barriers to working with autistic people such as a lack of evidence and training to guide their work and lack of connection with specialist services [21]. For autistic women, there is a specific lack of recognition of challenges that can present differently than in males which can also be the result of, until recently, failure to identify gender-specific presentations in autism (e.g. [22]). Camouflaging of autism symptoms is often associated with women [23].

These challenges can intersect with broader gender biases in healthcare, such as challenges in clinicians failing to attend to women’s report of physical symptoms and an over-reliance on attributing these symptoms to emotional causes (e.g. [24, 25]), resulting in a lack of both recognition of autism and the extent to which it may be impacting on autistic women’s experiences of health and mental health.

However, healthcare services are increasingly acknowledging the requirement to be aware of the needs of autistic individuals and to make adaptions to facilitate access and prevent harm. In the UK, the 2022 Health and Care Act established a requirement for health and social care staff to receive learning disability and autism training appropriate to their role [26]. Although research has explored ways to improve mental healthcare for autistic people, the effectiveness of these strategies is often uncertain due to low-quality or inconclusive evidence [27].

Within the UK National Health Service (NHS), the publicly funded healthcare system in Scotland, England, and Wales, community perinatal mental health teams (CPMHTs) in England provide support for women and birthing people experiencing moderate to severe or complex mental health difficulties during the perinatal period, defined as pregnancy through to two years postpartum. CPMHTs are multi-disciplinary and consist of psychiatrists, community psychiatric nurses, psychologists and psychological therapists, social workers, nursery nurses and peer support workers [28] and may also include occupational therapists, social workers and parent-infant therapists. Since 2016, following over £350 million investment by the English government, CPMHTs have been accessible throughout England [29].

Recent literature has increasingly examined the experiences of AWBP in the perinatal period. Studies have addressed specific aspects such as childbirth [30–33], breastfeeding [34], and the sensory dimensions of motherhood [35], as well as the perinatal period more broadly, encompassing pregnancy, birth, the postnatal phase, and interactions with healthcare services [1, 36–41]. Notably, studies have shown that AWBP are more likely to experience perinatal mental health problems, including higher rates of antenatal and postnatal anxiety and depression, than their non-autistic counterparts [1, 42, 43]. There are no studies around the prevalence of less common perinatal mental illnesses such as postpartum psychosis or perinatal obsessive-compulsive disorder (OCD) in AWBP. CPMHTs are therefore likely to encounter a disproportionately higher number of autistic individuals accessing their services, compared to the general population.

Until recently, both autism research and clinical practice have been gender-biased [44], with consequences of lack of recognition of autism in women before adulthood [45]. This means that CPMHTs may meet AWBP who lack a formal diagnosis of autism, including those who may not have considered it as a possibility, and/or have been misdiagnosed. CPMHT clinicians may face particular challenges around being both aware of signs and symptoms of autism as well as understanding how to support perinatal AWBP.

The current study therefore aimed to understand the skills, experiences and training of perinatal mental health staff in specialist Community Perinatal Mental Health Teams (CPMHT) in England in order to capture what is needed to provide appropriate support for AWBP with mental ill-health.

The research questions were:

i. What are the experiences of CPMHT staff working with AWBP?
ii. What barriers are there to providing good care and what adjustments can be made?
iii. How can CPMHT be improved to better meet the needs of AWBP?

## Methods

### Design and setting

This study employed a qualitative descriptive design [46]. A qualitative approach was chosen to provide rich information from individual reflections and views about skills, knowledge and experiences of working with perinatal AWBP in CPMHT.

The lead author (VW) is a lived-experience researcher who is autistic and accessed perinatal services. She designed, carried out the interviews and led the analysis and writing up. The rest of the study team included another researcher who is autistic who collaborated on the coding and analysis (CT), a non-autistic autism researcher (DC) and an academic clinical psychologist specialising in perinatal mental health (HOM). Together, our diverse experiences brought multiple perspectives. Additional input was sought from the autistic perinatal community: three individuals provided input on the plans to interview staff and specific comments on the interview topic guides.

The setting was four different CPMHTs in England. To leverage existing clinical research relationships, the sites were selected from those previously involved in the ESMI-II study: The EffectivenesS and cost effectiveness of community perinatal Mental health servIces project, which sampled ten CPMHTs across England representing variability in provision based on the project’s programme theory about what constituted good perinatal mental health care [47]. In this study the four sites were selected to optimise variability on staff and support provision associated with positive perinatal mental health outcomes. In our study, purposive sampling was used to obtain five different professionals from each CPMHT (twenty in total) – a psychiatrist, a psychologist, a practitioner holding a case-load (which might be a care coordinator, perinatal practitioner or community psychiatric nurse, depending on service terminology), an occupational therapist and another professional such as parent-infant therapist (a therapist who helps parents and babies develop positive relationships), peer support worker (a support worker with lived experience of perinatal mental health problems) or nursery nurse (a qualified childcare practitioner that provides specialist support to women and birthing people). This sampling strategy afforded a broad range of perspectives. Participants were not paid for their time as CPMHTs supported participation within their working hours.

The study was carried out in accordance with the standards of Good Clinical Practice and approved by the West of Scotland 3 Research Ethics committee (24-WS-0064) and the Southwest - Central Bristol Research Ethics Committee (19/SW/0218).

### Participant characteristics

Twenty people took part. From each CPMHT, a psychiatrist, psychologist, occupational therapist and practitioner holding a case-load were interviewed. In addition, a peer support worker, parent-infant therapist, pharmacist and nursery nurse participated. Five participants considered themselves to be neurodivergent, and seven said that they were not neurodivergent but had a close relative (e.g. child, sibling, parent) who was. Length of time working in CPMHTs ranged from 7 months to 11 years, with the median length of time being 4 years. Eighteen participants were female, and two were male; this gender-balance is typical of CPMHTs. All staff had received the Oliver McGowan mandatory training in autism. This is a government-backed program designed to ensure all health and social care staff in England receive appropriate training to support people with learning disabilities and autistic people, in line with their rights and needs [46].

### Data collection

Individual interviews were conducted between March 2024 and December 2024 by VW using recorded Microsoft Teams video calls. Written consent was provided by each participant on a consent form returned via email and study information and consent form reviewed at the start of the interview. Participants knew that the interviewer was an autistic mother who had previously accessed a different CPMHT. A semi-structured topic guide guided the interviews (see Appendix 1) Participants were asked for their number of years of experience working in CPMHT and if they thought themselves to be neurodivergent and if so, what type, as this was considered to be a factor that might influence the experiences shared (participants were not asked if they had a formal diagnosis). Interviews lasted between 25 and 77 min, with the median length being 39 min. VW filled out a reflexive diary after each interview was completed; this reflection enabled interview questions to evolve as interviews progressed, building on points raised by previous participants.

### Analysis

Interviews were transcribed, pseudonymised and uploaded to NVIVO for analysis. Participants were identified in the text by their professional role and a numerical identifier, except for the peer support worker, parent-infant therapist, pharmacist and nursery nurse who were referred to collectively as ‘other practitioner’ to preserve anonymity because only one for each of these professions were interviewed. Data analysis was conducted using reflexive thematic analysis, an approach that emphasizes reflective and thoughtful engagement with both the data and the analytic process [47].

All interviews were included in the analysis. VW immersed herself in the data through repeated reading of the transcripts to support a deep familiarisation. Initial coding began after the first four interviews, with codes developed inductively through close engagement with the data. These early codes were discussed with HOM, and the coding approach continued to evolve as further interviews were completed and analysed. CT independently read and coded 15% of the transcripts and offered interpretive insights, which contributed to the development of the coding process. Potential themes and sub-themes were generated by VW through an iterative and interpretive process and then discussed collaboratively with the team. The themes were refined in relation to the entire data set to ensure they captured shared patterns of meaning. Theme names were developed by VW to reflect the central organising concepts, with final themes reviewed and agreed upon by all authors.

## Results

**Fig. 1:**
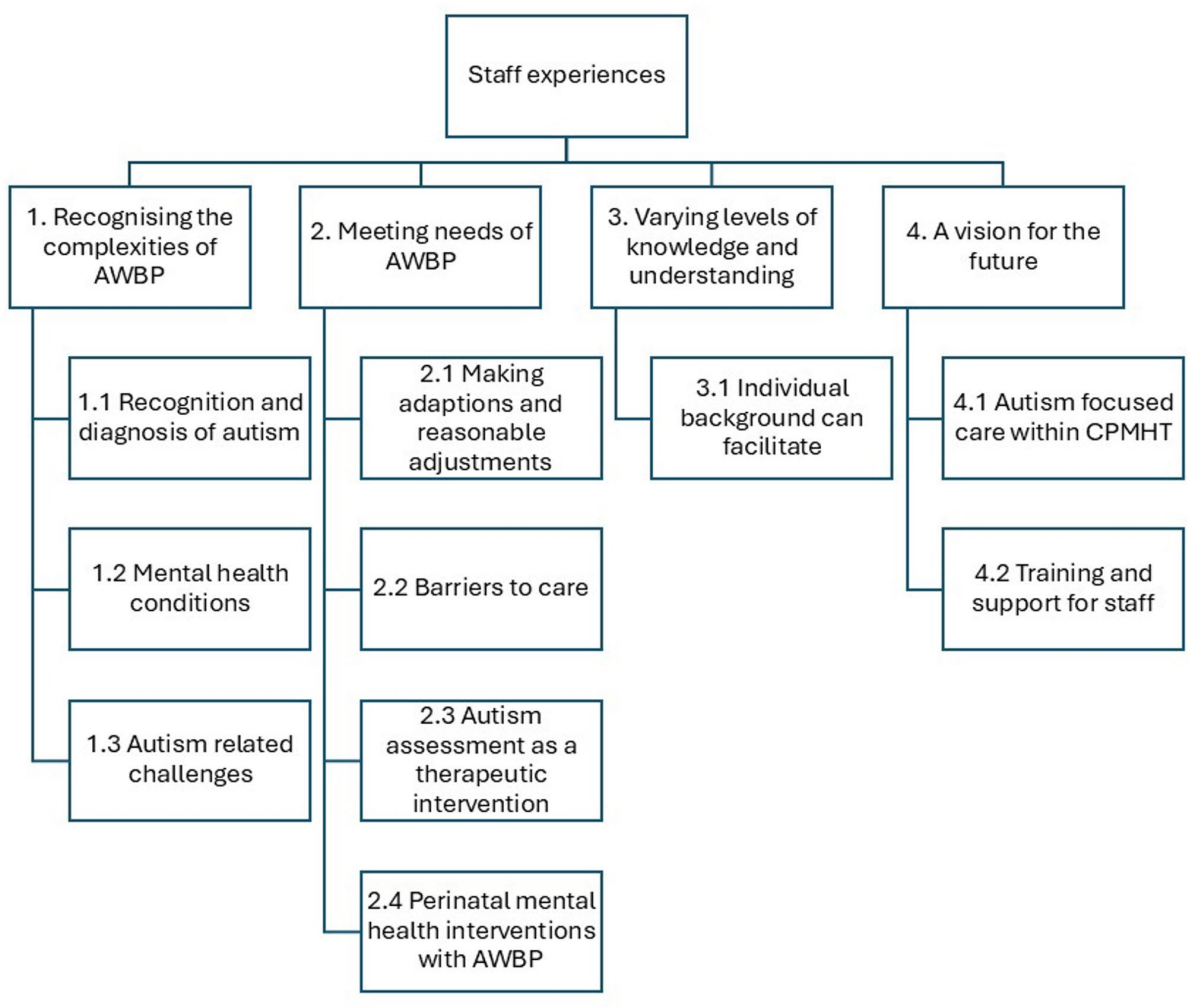
Themes identified in the interviews

Four major themes and seven sub-themes were identified. This is illustrated in Fig. 1.

### Recognising the complexities of AWBP

Clinicians described AWBP as presenting with multiple, intersecting challenges that CPMHT staff were required to navigate, including uncertainty around the recognition and diagnosis of autism, mental health difficulties and sensory and social factors shaping care.

#### Recognition and diagnosis of autism

Although some individuals had an existing autism diagnosis, staff reported that most had not undergone formal assessment prior to referral, leading to delays in identifying and planning for needs: “I don’t think I have actually worked with anybody that came into our team who had a diagnosis of autism to start off with” (Practitioner 3).

Several clinicians reflected that autism was often overlooked earlier in life, with many women misdiagnosed, commonly with emotionally unstable personality disorder (EUPD). CPMHTs frequently needed to disentangle these histories:

Sometimes it kind of baffles me that people have been under so many services in their past, because they’ve had so many mental health diagnoses kind of thrown at them, and then they come to us and it just seems very clear kind of where that those traits lie. (Psychiatrist 1)

Clinicians described the difficulty of differentiating autism from other conditions in the perinatal period: “…a lot of the challenges are around picking it up…rather than as part of a different presentation, you know, maybe EUPD or part of, you know, the normal challenges of parenting” (Psychologist 3).

#### Mental health conditions

Clinicians interviewed reported that the perinatal period seemed to be a trigger for mental health problems particularly for AWBP. For instance:

I see a lot of (autistic) women who…their lives have really been together and then have a baby, and it all falls apart, or it becomes overwhelming. And I wonder how many, in retrospect, of them, actually, where autism has played a part of that. (Psychiatrist 3)

Staff observed that, like non-autistic patients, AWBP presenting to CPMHT often experienced a wide range of mental health challenges including: anxiety and depression, for instance “she’s open to our team with anxiety and low mood” (Practitioner 1); birth trauma, for instance “she’d had a very, very traumatic birth” (Psychologist 3); “other trauma” (Practitioner 1), and perceived difficulties bonding with their baby, for instance mentioning “that emotional warmth that they hear other women describing, I don’t see them describing that. So they feel that something’s not right” (Psychiatrist 4).

#### Autism-related challenges

In addition to these problems, staff noted that AWBP frequently faced specific autism-related challenges. These needed to be understood in order to deliver effective mental health care: “their experience of being autistic is going to come into that sense-making a lot more than it might for someone who is not neurodivergent” (Psychologist 2).

Challenges were described as “unpredictability of routine” (Occupational Therapist 3), responding to change when “baby is constantly changing” (Occupational Therapist 4), “sensory overwhelm” (Psychologist 3), a “struggle to express their feelings” (Psychiatrist 4) and “normal coping mechanisms…they’re not accessible often when you have a baby” (Psychiatrist 3). There were also sensory challenges such as “the experiences in terms of physical experiences, of feeling pregnant” (Other Professional 1), accessing healthcare: “people being in close proximity to you, the unpredictability of appointments, waiting around in rooms with unfamiliar people” (Other Professional 1), physical touch from baby: “living …person on you all the time or breastfeeding” (Psychiatrist 2), noise: “babies are crying” (Psychologist 2), and weaning onto solids “the textures of foods and things like that” (Occupational therapist 2). Staff noted how it was critical to balance knowledge of these specific challenges in the context of mental health problems, noting that they often have a reciprocal and compounding impact.

### Meeting needs of AWBP

Clinicians interviewed described a range of ways in which they might meet the needs of AWBP. This is described in the following subthemes: making adaptions and reasonable adjustments, barriers to care, autism assessment as a therapeutic intervention and perinatal mental health interventions for AWBP.

#### Making adaptions and reasonable adjustments

Many staff were familiar with at least a basic level of some general aspects of autism and how they might impact AWBPs’ experiences of the perinatal period and their mental health. These staff described a range of general autism adaptions and reasonable adjustments that they might make, tailoring usual care to meet individual needs. One CPMHT had recently adopted a reasonable adjustments checklist, created by the local adult autism team, to provide a prompt for staff to think about potential adaptions around communication, arranging appointments and the environment. Sometimes practitioners asked individuals what they needed: “I ask them what (autism) means for them, what they find difficult, any adaptations that we need to make” (Psychologist 4).

One of the most common adaptions was flexibility around appointments. This included adapting the duration of the appointment, either to make it longer or shorter, or providing more sessions than usual for a particular intervention: “we needed to just go a little bit slower, for her specifically, to really be able to think about the material and…to ask questions” (Other Professional 3). Staff also described scheduling appointments at a regular time or providing all the dates for an intervention upfront, including holiday breaks, to enhance predictability. Several participants highlighted the importance of meeting at a location which met the client’s sensory needs, so appointments often happened in the client’s home. CPMHTs also offered alternatives to support groups, for instance by delivering one-to-one support sessions.

There were many ways staff adapted their communication with an autistic client. These included avoiding using the phone and using email or text messages instead:

(We offer) to change the kind of communication to what they preferred, so if it’s virtually, we can do it virtually, if it’s face-to-face they prefer, we’ll do it face-to-face, if they want to come into the office, that is also an option. (Practitioner 3)

Using practical demonstrations, drawing diagrams or providing written information to reinforce verbal communication was perceived as valuable. Some participants tried to avoid metaphors or checked for understanding.

Some adjustments were highlighted as being both useful to AWBP and to non-AWBP, for example offering home visits or providing summaries to aid memory. It was suggested that adopting autism-accessible practices offered benefits beyond those they were designed to support: “(they are) beneficial for every woman that we see, but especially beneficial for the people who are autistic, who we might not even recognise as being autistic” (Psychiatrist 4).

Although some staff members acknowledged the need to make adjustments for AWBP, the importance of individualising these adjustments was also recognized: “I think almost we would be doing people a disservice by having a blanket ‘this is what we do for autistic women’ …because what we’re doing is making an assumption” (Psychologist 2).

Several staff members underscored the importance of building a positive relationship as a key part of successful care for AWBP, noting how “just taking time to get to know that person” (Occupational Therapist 1) and their particular needs and approaches could support interventions that were relevant to their individual needs. Care was felt to work well when the individual practitioner had curiosity: “I think it’s just when I’ve been really thoughtful about what it means, so really curious about what does being autistic mean to this person? How does it come into their perinatal experience?” (Psychologist 2).

#### Barriers to care

Some participants noted that working with neurodivergent individuals was an unanticipated area of work for CPMHT and felt that AWBP were disproportionately represented in the caseload compared to the general UK population: “it’s one of those areas of perinatal work that has emerged as a really significant part of the, you know, presenting problems that we see, but that we didn’t really anticipate in advance” (Psychologist 3).

Without proper knowledge, skills or guidance in place, staff felt there were several barriers that prevented the delivery of good care to AWBP. Staff ability to communicate with clients who frequently found social communication and interaction challenging was perceived as a potential obstacle. Some participants reflected that they needed to communicate better what to expect before an appointment or check for communication preferences: “building up kind of an idea for them about how something might go beforehand, so thinking about what an area might look like or who - this is definitely something we can do better” (Occupational Therapist 4). One psychiatrist reflected: “we’re sometimes very vague in giving advice, and we need to be much more specific…Because if people are autistic, they crave that detail” (Psychiatrist 4). Written information could also pose a barrier: “it’s very much catered in the main for a neurotypical audience, and I think we’ve often been guilty of having ambiguous elements to our communication, which I think some of our autistic women would really struggle with, actually” (Psychiatrist 1).

Some staff felt that some of the usual interventions offered by CPMHT, across individual and group therapies, did not work as well with AWBP. This might be due to thinking styles: “my experience of mostly autistic women that I’ve worked with kind of struggled a bit more in psychology, maybe that it’s a bit more abstract” (Psychiatrist 3), or the requirement for social communication: “what we find is a lot of the autistic ladies just want to sit by the edge of the group, and don’t particularly want to sit in the circle and join in the conversation” (Other Professional 4). Staff might lack knowledge about how to individualise support: “it’s not like you can necessarily find a service that offers interventions that are necessarily designed for people with autism” (Occupational Therapist 4).

Some participants indicated how confusion about the roles of different services (i.e. mental health services versus autism-specific services) made it unclear who should support what problem and when:

[our] staff are sort of saying ‘What would be really helpful is some consultation with the autism team about how we can help this mum’, and the autism team are saying ‘Well, we actually haven’t diagnosed them yet, so we don’t really know them; we can’t offer you anything. (Psychologist 2)

Sometimes these difficulties had negative impacts: one staff member assumed that AWBP did not have psychiatric problems or that it was not the job of CPMHT to consider autism in this context: “we’re mental health, so that’s our focus” (Occupational Therapist 3). Another staff member described a client re-referred postnatally with severe depression after an antenatal referral was declined, as her original issue was framed mainly as autism-related: “she was probably suffering with quite significant anxiety, but because that label wasn’t stated, therefore, we didn’t accept [her]” (Practitioner 2).

Service barriers extended beyond CPMHTs. Several staff members reported that although they were able to work in an autism-friendly way and try to put in place adjustments, these were at times dismissed by other related services. For example, they would create a birth plan highlighting a client’s sensory needs that maternity services ignored:

we would have our own mental health kind of birth plan, but what we’re trying to plan for is somebody else’s environment…and there’s only so far maternity is geared to go, or equally has the understanding to go, to meet somebody with autism on the level which would be… catering to their needs (Other Professional 2).

Participants also described time constraints and space allocation limitations: “perinatal services are under so much pressure that it’s all good and well to say…[AWBP] might benefit from having a longer session… but we don’t have the time and the capacity to offer those things” (Psychologist 2).

The physical environment in which appointments could be offered was often inadequate as clinical space was limited, or staff did not have time to make home visits: “all the advice that you can give, ‘Oh, you know, plan your room and lighting this way’ and I’m lucky if I can get a room at all” (Psychiatrist 1).

#### Autism assessment as a therapeutic intervention

Full autism assessment was described as therapeutically important for undiagnosed clients that were likely to be autistic:

“[Our psychiatrist] has really been very thoughtful about how can we have some space to diagnose women as well, because there’s such massive waiting lists, and particularly if you’ve maybe spent a large chunk of your life believing that you have EUPD [Emotionally Unstable Personality Disorder], complex PTSD [Post Traumatic Stress Disorder], and then actually, you know, it might be autism” (Occupational Therapist 3).

However, the sampled sites varied in their ability to access assessment providers. In two sites, the consultant psychiatrist could provide assessment, although only one could do this as part of a multi-disciplinary team as recommended by the National Institute for Clinical Excellence (NICE) [34]. Elsewhere, long waiting lists exceeded the amount of time the individual might attend the CPMHT: “what we find is that they’re referred in, but we can often work with people for 18 months and they still haven’t got a diagnosis” (Psychiatrist 3). One participant reported: “the actual adult autism assessment team in our service are closed to referrals now because they’re just so swamped” (Occupational Therapist 1).

Other options were described, such as using formulation instead (i.e. the psychological approach to understanding a person’s psychological difficulties through assessment and integration of psychological theory): “formulation gives people enough holding to move through the service in a way that is manageable for them and has been adapted for them” (Psychologist 2). Another was to make a “working diagnosis” (i.e. a provisional diagnosis whilst awaiting further assessment): “I’m more keen to kind of go with a bit of a working diagnosis model, and be more confident in in saying to women, ‘Look, I think this is what the difficulties are’” (Psychiatrist 3). Sometimes self-diagnosis was acknowledged: “our encouragement is…if that’s a label that resonates with you, then act as if, because it can’t be a bad thing to do things that feel helpful” (Practitioner 2).

#### Perinatal mental health interventions with AWBP

CPMHTs used a range of approaches when working with AWBP, shown in Table 1.

 Occupational therapy was frequently highlighted as beneficial for AWBP because of its practical nature and helping adapt to life with a baby as an AWBP: “(Occupational therapy is) about routines, and if you’re autistic, you love a routine, don’t you? … All your routines have got broken by baby arriving, so you need to form all these new routines” (Psychiatrist 4). OTs who had received sensory integration training were felt to be particularly helpful given the impact of sensory challenges of having a new baby “we’re really, really lucky to have in our team a wonderful occupational therapist… and she does a lot of sensory assessments with women” (Psychologist 1).

**Table 1:** Interventions mentioned as being used with AWBP (grouped according to interventions commonly offered by CPMHT in England)

Occupational therapy interventions	Sensory work such as Sensory Integration Therapy (used to help to cope with challenges processing sensory input) (Occupational therapist 4) or Sensory Ladder (a tool to help people understand their sensory experiences and regulate their emotions) (Occupational Therapist 3)
	Practical skills work such as preparing food for baby (Practitioner 3), taking baby out using public transport (Occupational Therapist 4) or developing routines (Occupational Therapist 2)
Parent-infant interventions	Video Interaction Guidance (an intervention where the parent is guided to reflect on video clips of interactions with their baby) (Psychiatrist 1, Practitioner 1)
	Circle of Security (a parenting programme supporting parent-child relationships) (Other Professional 3)
	Understanding and responding to baby cues (Practitioner 1)
Psychological therapies	Dialectical Behavioural Therapy skills group (Psychiatrist 1)
	Creative therapy such as journalling or art therapy (Psychiatrist 1)
	Compassion focussed therapy (Psychologist 3)
Working with partners/family	Providing psychoeducation about autism and how to support their partner/family (Occupational Therapist 4)
Medication	Medication to treat mental health conditions (Psychiatrist 1, Psychiatrist 2)
Birth planning	Developing a birth plan to be shared with maternity services (Psychologist 1, Occupational Therapist 1).

### Varying levels of knowledge and understanding

Knowledge and understanding of autism varied both across CPMHTs and individual clinicians. When CPMHT highlighted the needs of AWBP at the service level, more individual staff members were likely to understand and report responding to these needs: “we’ve got a special interest group: we’ve got a working group, we’ve got two autism champions” (Psychiatrist 1). Staff who felt more confident were able to reflect on instances where their practice had fallen short and communicate this honestly to patients “So I’ve got it wrong a few times, but whenever I do, I actually, I start off with an apology, saying, ‘If I get any of this wrong, please just tell me, because I’m learning and I need to get better, and I might have misunderstood’” (Psychiatrist 1).

However, in another service, one participant expressed a lack of knowledge of what to do and a sense of poor efficacy about being able to explore options: “I would say that I probably haven’t, if I’m being honest, done anything specifically different (for AWBP)” (Other Practitioner 1). Sometimes a staff member might not feel confident, especially if they lacked training: “I can sometimes feel like I might be letting a woman down…just having to do something in a bit of a different way that I don’t know is necessarily the right way” (Occupational Therapist 4).

#### Individual background can facilitate

The participant’s background of personal experience, work experience or professional training often enabled them to work effectively with AWBP. Some of the neurodivergent participants or those with neurodivergent family members recognised that the personal knowledge arising from their own experiences was an advantage: “I get what they’re going on about, and they get me” (Psychiatrist 4).

Several staff had previously worked in an autism service, and this provided specialist knowledge and interest that others did not have. Many staff highlighted the value of working as part of a team when working with an autistic individual, giving opportunities to discuss a client or to draw on the knowledge of different professions: “I think expertise within the team…is really positive” (Other Professional 3). Occupational therapists often, but not always, seemed to have a good level of understanding of autism needs, often taking a lead on autism planning: “I think because there’s a practical element, the sensory element, it really just makes sense, I think, for OTs to…lead on this and be …the experts” (Occupational Therapist 3).

### A vision for the future

Participants were asked to consider how CPMHT could best support AWBP if they were given unlimited resources. This fell into two subthemes: autism-focused care within perinatal health, and training and support for staff.

#### Autism-focused care within CPMHT

Most participants wanted to see a specific focus on autism in their service, to meet autistic needs alongside a client’s perinatal mental health needs. Some aspirations for care were shaped by service design and inter-agency collaboration, such as the desire for faster assessments to enable earlier autism diagnoses: ‘…so people were getting diagnosed as autistic sooner’ (Psychologist 4); “post-diagnostic support, and a real sort of understanding of what this is that they’ve just been diagnosed with” (Psychiatrist 3); an autism pathway, “if you are neurodiverse, this is the way it can work, this is how it can look. This is the journey, this is what we can offer” (Other professional 1) and “really good joined-up care with midwifery” (Psychiatrist 3). Other hopes related to greater OT provision “it would be amazing to have another OT [to] just be more dedicated to this work, you know” (Occupational Therapist 3) which might enable more sensory support such as “sensory profiling for every woman” (Occupational Therapist 4). Another goal was “peer groups for all the autistic mums in our service” (Psychologist 2) and psychoeducation for partners “their partners might need some more education around that diagnosis, that would be something that I think would be nice for our kind of service to also include” (Practitioner 3). Another desire was for the development of evidence-based adaptions to therapy for AWBP: “we’ve got specific…interventions, how do we modify them to make them autism-informed?” (Psychiatrist 1).

Ideas for staffing included adding a specialist autism practitioner to the team or employing an autistic perinatal peer support worker. One participant highlighted the need for “having specific practitioners who, if we’ve got a woman whose challenges with her mental health in the perinatal period are quite intertwined with her experience of being autistic, then she is held by this specialist practitioner” (Psychologist 2). An autistic peer support worker could both provide support to individuals but also challenge the system from within: “Because they’re in all our meetings…They can help us all work out what we’re doing wrong” (Psychiatrist 4).

Some participants considered adaptions that could be made to the existing service. Some were relatively inexpensive, such as “sharing videos with people so that they know what to expect in visits when they come” (Practitioner 1) or improving printed literature “we have lots of kind of printed leaflets about the medicines, but they’re not necessarily autism-friendly” (Psychiatrist 4).

Several participants highlighted the need for future service development to be co-produced: “I think that could revolutionise the way that we provide service, because obviously it wouldn’t be me, as an obstreperous male saying it, it’s actually, ‘This is a service user saying this and actually could we just listen to them?’” (Psychiatrist 4). Staff also emphasised the need to have the support for the time necessary to implement adaptations “it is about resources: like, when do we fit that in, how can we make sure that we prioritise that among everything else?” (Occupational therapist 3).

Two CPMHTs were already developing their vision for AWBP. An autism group about to be implemented by one CPMHT was described as “a safe and supportive environment for autistic mothers to connect, share experiences and receive peer-led and professional-led support” (Occupational Therapist 3). Another CPMHT had appointed an autism champion: “within our team I’m an Autism Ambassador” (Practitioner 1).

#### Training and support for staff

Staff consistently highlighted the need for better training and supervision to effectively support AWBP. They specifically emphasised the importance of autism training tailored to the perinatal focus of CPMHT: “you could have training in autism, but then sort of more specific than that: autistic parents” (Occupational Therapist 1). Staff also expressed a desire for training in evidence-based interventions for AWBP, such as sensory integration. Finally, they recommended strengthening connections with autism teams for supervision and knowledge sharing: “there could be a regular space…between the autism service and perinatal, to be thinking about women, how we can support them” (Psychologist 1).

## Discussion

This study provides the first account of staff perspectives on supporting autistic women and birthing people (AWBP) within community perinatal mental health teams (CPMHTs). While existing research has established that AWBP are more likely to experience perinatal mental health problems [1, 42, 43, 48, 49], previous studies have focused largely on the perspectives of AWBP and their interactions with maternity services more broadly, rather than with specialist perinatal mental health services. Given the increased likelihood of perinatal mental health problems, AWBP are likely to be over-represented in CPMHTs. One small study interviewed five AWBP who accessed a CPMHT [50] but no studies have interviewed staff from a CPMHT. Our findings therefore extend existing literature by identifying the barriers staff face, the adaptations they are making, and the priorities they see for future service development.

We found that while CPMHTs recognised the importance of adapting care for AWBP, staff often lacked clarity about how best to do so. Although some innovative practices were emerging such as running groups specifically for AWBP, most staff members reported limited information on the specific needs of AWBP and on which approaches were most effective. Identified challenges included barriers to delivering appropriate care such as the physical environment, recognising undiagnosed autism in the perinatal period, and developing appropriate interventions. Staff highlighted training, autism-specific peer support, and co-production as key priorities for service development.

Our findings reveal multiple barriers that perinatal mental health providers face in delivering effective and inclusive care to autistic individuals. This aligns with the evidence that autistic women find it difficult to access healthcare during the perinatal period found by Elliot et al. [9] and our previous study [6]. These barriers to care reflect those commonly reported by healthcare professionals working with autistic adults and align with broader literature documenting the challenges autistic individuals face in accessing both mental health and general healthcare services. These include communication challenges with autistic patients, meeting individual sensory needs, limited autism knowledge and training among professionals, and systemic issues such as time and resource constraints or lack of collaboration between services [18, 19, 21].

Autism diagnostic assessment was a particular concern. Staff reported that many AWBP were suspected to be autistic but had not been formally assessed. This reflects wider pressures on diagnostic pathways in England, where long waiting lists persist despite NICE guidance that assessments should be completed within 13 weeks [51]. By September 2024, more than 180,000 people had been waiting longer than this, with staff in our study anecdotally reporting waits of several years. Although some psychiatrists were able to assess and diagnose patients within CPMHTs, others did not, and many professionals expressed limited confidence in identifying autism, particularly in the perinatal context.

Despite these challenges, staff described some effective adaptations that improved accessibility, although these were not always perinatal specific. Examples included choosing appropriate venues for appointments, scheduling sessions at the same time each week, and using alternative communication methods such as providing email summaries. These align closely with Doherty et al.’s Autistic SPACE framework, which promotes service adaptations to improve accessibility for autistic adults [52]. Staff also reflected on therapeutic approaches. While a range of therapies were used with autistic patients, many participants felt that the practical strategies offered by occupational therapy were particularly beneficial, as they focused on building routines and addressing sensory needs. However, we do not know of any research that examines the role of occupational therapy either within perinatal mental health or with autistic adults more broadly. Overall, participants’ emphasis on individualised care reflects previous recommendations from research on autistic mental healthcare [53, 54], while underscoring the importance of tailoring these approaches to the perinatal context.

Our study suggests several areas for service improvement. A common refrain was the need for targeted training that addressed the intersection of autism and perinatal mental health, as generic autism training, though now mandatory within the NHS [55], was seen as insufficient for meeting the specific needs of AWBP. However, although staff felt that specific training would build confidence, alone it is unlikely to be sufficient. Barriers such as limited time and resources, commissioning structures that prioritise mental health over neurodevelopmental needs, and lengthy waits for autism diagnosis, all constrain staff capacity to adapt care. Another area of development highlighted was the use of peer support. While McLeish et al. [56] describe both its benefits and drawbacks of within CPMHTs, this approach has not yet been examined for autistic perinatal patients. However, initial research into the value of peer support for autistic adults is promising [57]. Participants also emphasised the importance of co-producing service development with autistic people, recognising them as experts in identifying what might work best. Co-production is increasingly valued within the NHS as a mechanism for improving service quality [58] and could provide a vital means of ensuring that CPMHTs meet the needs of AWBP. However, to be implemented meaningfully, both peer support and co-production require adequate resourcing.

One limitation of this study is that staff members either self-selected to participate or were nominated by a senior team member because they were likely to be interested in taking part or were thought to have relevant experience. This may mean that participants might have a particular interest in autism, potentially not reflecting the views of those with less interest and knowledge amongst the team. In addition, the focus on the core professionals of CPMHT (Psychiatrist, Psychologist, and Community Psychiatric Nurse (CPN)/care-coordinator) alongside Occupational Therapists means that the diversity of professional experiences in CPMHTs may not be fully reflected. This was mitigated by asking each service to nominate another kind of professional to take part, providing voices of peer support worker, parent-infant therapist, nursery nurse and pharmacist. Moreover, this study only investigated CPMHTs in four NHS trusts across England (out of a total of 223 trusts) and findings may not be generalisable to other UK nations and other countries. Although this is a model of care specific to the UK, other countries such as Ireland, Germany and the United States do provide outpatient care for those with perinatal mental health conditions (59) and some of the learning may be transferable. Further research could examine more in depth the specific value of occupational therapy within CPMHTs for AWBP.

## Conclusions

This study found specific barriers and challenges experienced by staff within perinatal mental health services that might prevent appropriate mental health support to AWBP. We found that multidisciplinary mental health teams could benefit AWBP by delivering individualised care and occupational therapy is particularly seen as valuable for these clients. Notable differences in knowledge and understanding were evident, not only between individual staff members but also across the different CPMHTs involved in the study; this suggests a lack of specific training and systemic support although it also reflects the value that lived experience of neurodivergence could bring to staff members.

When thinking about future improvements, staff expressed a clear demand for the development of autism-informed care pathways, underscoring the current gaps in service provision and highlighted the need for targeted, practical training to equip them with the necessary skills and confidence. Additional areas of focus could include the appointment of autism champions and the integration of autistic peer support workers, which could enhance the relevance of care. Equally important is the meaningful involvement of individuals with lived experience of autism and perinatal mental health challenges in the co-production of service development.

Our study highlights the distinctive structure of UK CPMHTs and the specific support they provide to AWBP experiencing moderate to severe perinatal mental health problems. The multidisciplinary nature of these teams presents a valuable opportunity to examine how various forms of support interact and contribute to outcomes for AWBP. However, further research is needed to critically assess the transferability and effectiveness of this model in other contexts, for example to services for individuals with mild-to-moderate perinatal mental health needs, or in international settings where healthcare systems and cultural understandings of autism may differ.

## Supplementary Information

Supplementary Material 1.

## Data Availability

The data that support the findings of this study are not openly available due to reasons of confidentiality and are available from the corresponding author upon reasonable request.

## Abbreviations

AWBP: Autistic women and birthing people
CPMHT: Community perinatal mental health team
CPN: Community Psychiatric Nurse
EUPD: Emotionally Unstable Personality Disorder
NHS: National Health Service
NICE: National Institute for Clinical Excellence
OCD: Obsessive compulsive disorder
PTSD: Post Traumatic Stress Disorder
