# Supplementary material for: “I ask them what autism means for them”: a qualitative study of staff experiences of working with autistic women and birthing people in community perinatal mental health teams
Source: BMC Psychiatry. 2025 Oct 27;25:1024. doi: 10.1186/s12888-025-07497-6 (PMC12560391; doi:10.1186/s12888-025-07497-6)
Supplement: Supplementary file 1 — Supplementary Material 1. [file 12888_2025_7497_MOESM1_ESM.docx]

# **Appendix 1**

Questions we asked each participant during the interviews:

- Tell me about your role and what you do on a day to day basis.
- What difficulties do you see in autistic women in the perinatal period?
- What do you think autistic women with mental health problems and their babies need in terms of support and treatment in the perinatal period?
- What does the delivery of good care to autistic women look like to you?
  - What impedes this? What practical solutions could you find?
- What elements of the service are useful to women and their babies?
- How do you maintain engagement of women?
- What do you find the most effective communication style with autistic women?
- Do you make any adjustments with your practice for autistic women?
  - Structure/predictability; allowing more time/continuity; pharmacotherapy; sensory awareness
- Do you have an idea of how many autistic women you see in your service? Has this increased since the service opened? How many of these women are undiagnosed?
- Have you had any training around working with autistic women in the perinatal period?
- What type of supervision is provided for working with autistic women?
- What impact do you think the service has on autistic women?
  - How does that impact on their outcomes?
- Can you think of a time when you have worked with an autistic woman and it has gone well and tell me why it went well?
- And conversely, can you think of a time when you worked with an autistic woman and it did not go so well and think about why?
- Although this may vary, could you tell me what happens to autistic women who are discharged from the service?
  - Is there anything you put in place specifically for this group of women?
- Are there other services that you work with when working with an autistic woman?
  - Probe – specialist autism/LD teams, HV, midwives, external agencies
- When it comes to maternity care, what adjustments are you able to put in place for women? Do you work with specialist midwives to implement these?
- Imagine that we were able to create a perinatal service that really met the needs of autistic women. What do you think would be important?
